# Recent Advances in Laparoscopy

**DOI:** 10.3390/jcm10010131

**Published:** 2021-01-02

**Authors:** Ibrahim Alkatout, Matthias Biebl

**Affiliations:** 1 Department of Obstetrics and Gynecology, University Hospital Schleswig-Holstein Campus Kiel, Arnold-Heller-Str. 3, 24105 Kiel, Germany; 2 Department of Surgery, Campus Virchow Klinikum, Charité–Universitätsmedizin Berlin, Augustenburger Platz 1, 13353 Berlin, Germany; Matthias.biebl@charite.de

At the end of 2019, we received reports of abnormally high rates of severe pneumonia and mortality in a city named Wuhan in the province of Hubei in China. The reports reached Europe and Germany, and the rising number of infections became an impending threat to public health on a worldwide basis. More than 400,000 cases of the disease and more than 18,000 deaths were reported in March 2020. A novel form of the coronavirus known as “severe acute respiratory syndrome coronoavirus-2 (SARS-CoV-2)” was responsible for a disease complex referred to as the coronavirus disease 2019 (COVID-19). The virus reached Germany as early as 27 January 2020. Despite initial hopes of being able to curtail the problem, private and professional lives were severely locked down due to COVID-19, which evolved into a worldwide pandemic and a most serious threat to global health within a few months [1].

The pandemic had far-reaching effects on personal and economic lives in Europe and throughout the world. One of the first consequences in surgery was the postponement of elective procedures. The numbers of patients admitted for surgery in hospitals were reduced to a minimum, and the resources of emergency care units were maximized to provide sufficient care for patients with the new disease.

An invitation from the Journal of Clinical Medicine to release a special issue on Recent Advances in Laparoscopy was received exactly during this time period. To quote the erstwhile British Prime Minister Sir Winston Churchill: “Never let a good crisis go to waste.” The unprecedented crisis of a pandemic became the nascent hour of this special issue. Although many researchers were preoccupied with several matters other than academic paperwork, we pursued the formidable task of wrapping up and presenting the last decade of surgical progress in appropriate form.

The name coined for the special issue was Recent Advances in Minimally Invasive Surgery. Both editors of the special issue are aware of the fact that minimally invasive surgery encompasses the entire field of surgery. Since we serve a gynecological surgeon as well as a visceral surgeon in Europe, this issue is focused on the story of minimally invasive surgery in these two fields. A great deal has happened in both sectors. The aim of the minimally invasive surgeons is, and always has been, to reduce the trauma of surgical access for the patient. In patients with thoracic or abdominal pathologies, the surgical access should provide vision, access to the field of surgery, sufficient working space for safe dissection, and—in cases of resection—the ability to remove the specimen through the access route. Given the skills of several generations of surgeons in open surgery, it became clear that the reduction of surgical access trauma could only be achieved by consistent improvement of surgical instruments, paired with profound knowledge of anatomy and standardized procedures. Another fundamental prerequisite would be a transformed mindset towards surgery as such, and the ability “to think outside the box.” We are faced with the challenge of finding new solutions to old problems.

Since ancient times, medical practitioners wished to inspect the insides of the human body in order to understand its complexity and treat diseases effectively. Easily accessible body cavities, such as the mouth, rectum, or vagina were inspected in ancient times with the aid of speculums. The origin of endoscopy can be traced back to a reference in the Babylonian Talmud. The treatise describes a lead funnel with a curved mouth, furnished with a wooden outlet (Mechul). The origin of minimally invasive surgery is largely associated with Philipp Bozini, who died in 1809 at the young age of 36 years. His innovative approach resulted in the gift of light conductors to the medical community, which permitted the investigator to view the body through an endoscope. The journey that followed was a challenging one. Georg Kelling performed the first endoscopic procedure. He viewed the stomach of a dog using Nitze’s cystoscope and an air insufflation apparatus at the Natural Scientists’ Meeting in Hamburg, Germany, in 1901. The history of laparoscopy and its introduction in surgical practice is a story of many researchers and pioneers who, for many years, battled against prevailing opinion and confronted rejection of their brainchild: their vision of performing “gentle operations”. Many of these pioneers were ignored, shunned as dreamers, or even considered insane [2]. 

An interesting characteristic of minimally invasive surgery is that its evolution was never linear. It was by no means similar to oncologic surgery, which followed the familiar academic path of introducing new treatments through formal evaluation in a prospective study environment. Almost every breakthrough or innovation in minimally invasive surgery was initiated by a few innovators, picked up enthusiastically by a select group, and then disseminated to others. Subsequently, the innovations were evaluated carefully in a formal setting and incorporated definitively into the medical armamentarium. This problematic evolution *per se* was further aggravated by the medical technology industry, which developed new devices but promoted their dissemination in the interests of profit rather than patient benefit [3,4].

Over the past decades, this evolution was accompanied by profound changes in oncologic principles during the last few decades. It led to a refinement of surgical techniques as well as the extent of resection. Through a meticulous scientific approach and suitably designed trials, the medical community worked diligently to establish reasonable standards. Simultaneously, ongoing specialization in the field of surgery has demonstrably improved the quality of patient care. In addition to organ-oriented specialists, we now even have disease-oriented specialists. Both of these have clearly replaced the traditional distinction between a medical doctor and a surgeon, as we knew them fifty years ago. However, innovative surgeons who tried to introduce new ideas were bitterly opposed by an academic community focused on creating their own standards based on proven and established principles of long duration. Until recently, the section of minimally invasive surgery at many surgical departments in Europe was an ill-defined mixture of whatever the hospital had to offer by way of appendectomy, hernia surgery, bariatric and reflux surgery, and selected procedures in colon surgery.

Fortunately, the situation changed very profoundly for the better over the last decade. All of the above mentioned subspecialities have—albeit reluctantly in some cases—adopted the existing minimally invasive techniques in their respective fields. These procedures have fully arrived in several major academic centers worldwide. It was a much desired and urgently needed step forward. The academic force of a well-connected international medical community is a prerequisite for the timely development, evaluation, and dissemination of new techniques. Based on the notion of reducing access trauma, the innovators had to (a) balance the new techniques against evolving oncologic standards, and (b) realize that no subsequent measure to reduce access trauma could be as impressive as the initial departure from open surgery in favor of the minimally invasive approach. 

Consequently, not all techniques stood the test of time and not all promises could be fulfilled. Single-port surgery created a stir in the medical community more than a decade ago [5], but has long descended into the assortment of several existing but meagerly utilized techniques. The purpose of NOTES (natural orifice transluminal endoscopic surgery) [6] is to perform surgery without leaving any visible scars, but the procedure has almost disappeared after more than ten years of eager innovation. However—and this seems to be another unique aspect of the evolution of minimally invasive surgery—virtually every technique, invention, and new approach left its footprint in the evolution of surgery even after the initial concept had been abandoned [7]. Both, single-port surgery and NOTES paved the way for a novel type of pelvic floor surgery [8,9,10]. Transanal access routes were also subject to the rise and fall of new and thrilling techniques. The role of these access routes in specialized surgery for low rectal cancers is yet to be defined. Robotic surgery—designed as a means of remote access to medical care on a worldwide basis—has evolved through several generations of technical advancement. Robotic surgery has demonstrably revolutionized the precision of surgery, and also promises to achieve a hitherto unprecedented improvement in the outcome of treatment for patients [11]. Randomized controlled trials will be needed to prove this fact in the clinical setting.

Revolutionary perioperative treatment algorithms such as fast track and enhanced recovery after surgery (ERAS) have shown how much needs to be done around the operating room in order to optimize patient care. This gave rise to the rather puzzling situation of fewer complications and more favorable recovery in patients undergoing open surgery with optimized perioperative treatment compared to those who underwent minimally invasive surgery without an appropriate environment. Besides, it hindered the translation of reduced operative trauma into measurable patient outcome parameters such as the length of hospital stay or postoperative recovery in patients undergoing extensive cancer surgery, including esophageal resection. Again, it became clear that surgery is one instrument in the “concert” of patient care. No expert can play alone. This became even more evident after the advent of complex and highly successful medical cancer treatments with staged, perioperative, and truly multimodal treatment algorithms. The current task of oncologic surgery is no longer a “once in a lifetime” chance to “get rid” of the tumor. Rather, it is a module in modern cancer care that can be used repeatedly and also must be integrated into the mosaic of ongoing multidisciplinary treatment. This—together with the optimization of perioperative care—will be the true challenge of minimally invasive surgery in the coming decade [12]. 

Therefore, this issue of the Journal is not only focused on the winners of widespread medical attention such as robotic surgery, but also provides a platform for some of the lesser known advances, techniques, and sophisticated surgical solutions in gynecologic and visceral surgery [13,14]. Furthermore, we have tried to shed light on questions concerning the implementation and appropriate teaching of new techniques [15], in addition to flanking solutions aimed at improving perioperative patient care.

The common goal of this collection of medical studies is to present the various elements of a rather difficult symbiosis of technical progress, industrial participation in healthcare, medical knowledge, and global data exchange. 

With this approach, the authors express the hope that every medical obstacle between China and Germany will be overcome, and will prove surmountable in current times as well as in the future. 
